# The Effects of Ketogenic Diet Treatment in Kcna1-Null Mouse, a Model of Sudden Unexpected Death in Epilepsy

**DOI:** 10.3389/fneur.2019.00744

**Published:** 2019-07-10

**Authors:** Yandong Ren, Jinlong Chang, Chengchong Li, Cuicui Jia, Ping Li, Yuhua Wang, Xiang-Ping Chu

**Affiliations:** ^1^School of Mental Health, Qiqihar Medical University, Qiqihar, China; ^2^Department of Biomedical Sciences, School of Medicine, University of Missouri-Kansas City, Kansas City, MO, United States

**Keywords:** ketogenic diet, epilepsy, sudden unexpected death in epilepsy, Kv1.1 knockout mouse, gender, age

## Abstract

Sudden unexpected death in epilepsy (SUDEP) is a leading cause of abrupt death in patient with epilepsy. It represents 5–30% of all rapid deaths in individuals with epilepsy. Ketogenic diet (KD) has been used in clinic for treatment of epilepsy for many decades. However, the cellular and molecular mechanisms underlying the SUDEP and the relationship between KD and SUDEP remain uncertain. Kcna1-null (Kcna1^−/−^) mouse, an animal model of SUDEP, is frequently used to study mechanisms underlying SUDEP. The current mini-review focus on risk factors for SUDEP and their relationship with KD treatment in Kcna1^−/−^ mice. Emerging data suggest that factors including seizure frequency, longevity, rest, age, and gender both in Kcna1^−/−^ mice and KD treated Kcna1^−/−^mice are involved in SUDEP. This provides valuable prediction for clinical application of KD for treatment of SUDEP.

## Introduction

Epilepsy is a common neurological disease defined by recurrent seizures (1). The typical cause of death associated with epilepsy is sudden unexpected death in epilepsy (SUDEP) (2). It is characterized as the rapid, unanticipated, witnessed or unwitnessed, a non-traumatic, non-drowning death that occurs in benign circumstances in an individual with epilepsy in a reasonable state of health, in whom postmortem examination does not display an alternative structural or toxicological cause for death (3). The incidence rate of SUDEP is approximating 1 in 1,000 cases every year (4, 5). There are several risk factors associated with SUDEP including generalized tonic-clonic (GTC) seizure frequency, postictal generalized electroencephlogram suppression, lower intelligence quotient, cardiac arrhythmias, respiratory dysfunction, gender, early age at seizure onset, and polytherapy with antiseizure drugs (ASDs) (2, 6–8). Recent studies also demonstrate that genes are also associated with SUDEP. It includes KCN (A1, Q1, and H2), SCN (1A, 2A, 5A, and 8A), DEPDC5, HCN2, RYR3, and HTR2C. They are potential risk factors for SUDEP (8–10).

The high-fat, low-carbohydrate/protein ketogenic diet (KD) is recognized as an effective, non-surgical treatments for refractory epilepsy (11). KD treatment for epilepsy has been recorded since the fifth century and reported in scientific literatures since the early 1900s. Patients with epilepsy are strongly accept the KD therapy (11).

The Kcna1-null (Kcna1^−/−^) mouse, deleting the voltage-gated potassium channel α subunit Kv1.1 (12), exhibits severe spontaneous recurrent seizures (SRS). The Kcna1^−/−^ mice reveal early seizure in postnatal development and display premature unexpected death (13). It serves as a valuable animal model for SUDEP research.

Although studies have been confirmed the anticonvulsant efficacy of KD in epilepsy, the underlying mechanisms and risk factors in Kcna1^−/−^ mice with KD treatment remain unsolved. Despite lack of direct clinical evidence about the efficacy of KD in SUDEP in Humans, KD reveals an effective treatment for refractory epilepsy. It can reduce seizure frequency (14), decrease seizure severity, and improve cognitive ability (15), cardiac dysfunction and quality of life (16). Thus, the KD appears to be highly effective in treating refractory epilepsy. The role of KD in SUDEP is becoming better understood with Kcna1^−/−^ mouse as a model of SUDEP (13, 17, 18). The current review will concentrate on recent studies of KD treatment and mechanisms in Kcna1^−/−^ mice. The effects of risk factors such as seizure frequency, longevity, rest, age, and gender in Kcna1^−/−^ mice and KD treated Kcna1^−/−^ mice are discussed.

### Kcna1-Null (Kcna1^−/−^) Mouse as a Model of SUDEP

Kcna1-null (Kcna1^−/−^) mouse is the mouse of disruption of the voltage-gated potassium channel alpha subunit (K_V_1.1). It reveals frequent spontaneous seizures throughout their adult life (18, 19). These null mice are widely used to explore potential genetic and pathophysiological mechanisms of SUDEP (19). The model displays many similarities with human SUDEP risk factors. These similarities include (1) young age; (2) seizure frequency; (3) long duration of seizure, (4) GTC seizures; (5) early onset seizure; and (6) seizure-evoked bradycardia and asystole progressing to cardiac arrest (12, 17, 19–22).

### Seizure Frequency in Kcna1^−/−^ Mouse

Higher seizure frequency reveals a negative influence on the quality of life in patient with epilepsy. Recent studies have shown that seizure frequency is associated with age in Kcna1^−/−^ mice. They found that seizure frequency increases with age until sudden death in Kcna1^−/−^ mice. Before postnatal day 21 (P21), behavioral seizures cannot be detected in Kcna1^−/−^ mice. The seizure numbers in Kcna1^−/−^ mice increase gradually after P21 and seizures persist until death with an average age of P43 (17). However, the severe seizure account for all seizure number remains similar throughout life and usually occurs when it approaches death in Kcna1^−/−^ mice (5, 12, 17).

### Rest Deficiency in Kcna1^−/−^ Mouse

Rest time in mouse, detected by continuous infrared telemetric actimetry and switch-closure activity monitoring, stands for sleep time in mouse, it's deficiency is a risk factor in seizure and also associated with Kcna1^−/−^ mice (18). Studies have shown that compared to wild-type (WT) mouse, the rest time is similar at P26, but there is a prominent reduction with age in Kcna1^−/−^ mice, beginning at P30. When compared to their youngest age, the rest time of Kcna1^−/−^ mice is decreased by 32% at P42 and by 47% at P50, respectively (18). The data reveal that Kcna1^−/−^ aged mice display a reduction in rest time.

### Possible Mechanisms of SUDEP in Kcna1^−/−^ Mouse

Kcna1^−/−^ mouse usually has a severe seizure phenotype with myoclonic and GTC seizures (17). These seizures process can be divided into three steps. First step, tonic arching and tail extension. Second step, rearing and forelimb clonus. Third step, generalized synchronous forelimb and hindlimb clonus (23). Kcna1^−/−^ mouse also experiences premature death, which may be due to cardiac and/or respiratory dysfunction associated with severe generalized seizure activity (17, 18, 23). Hippocampus might play a crucial role in seizure pathogenesis for Kv1.1 protein, which has a high expression in hippocampus especially in the CA3 and dentate regions (24). The amygdale and other limbic circuits might also be involved in seizure generation or spread in Kcna1^−/−^ mouse (25).

### Pathophysiological Mechanism in Kcna1^−/−^ Mouse

As an efficient SUDEP model, Kcna1^−/−^ mice has been widely used for study the pathogenesis of SUDEP in epilepsy (12, 21, 25–28). In previous studies, cardiac and respiratory dysfunction was considered to be the main cause of SUDEP in Kcna1^−/−^ mice (12, 26, 28). Cardiac abnormalities such as bradycardia, premature ventricular contractions, and atrioventricular conduction blocks were increased dramatically in Kcna1^−/−^ mice as compared with WT mice (12). Respiratory failure such as hyperventilation, tachypnea, hypopnea, bradypnea, and apnea were observed in Kcna1^−/−^ mice during severe seizures that may result in sudden death (26). The respiratory disorder always preceded cardiac abnormalities in Kcna1^−/−^ mice during spontaneous convulsive seizures. Therefore, the respiratory dysfunction has been considered the main driver of cardiac dysfunction in Kcna1^−/−^ mice and occurred much more common than cardiac dysfunction during seizures underlying SUDEP risk (28).

Kv1.1 alpha subunit protein is mainly expressed in the brain, but it reveals lower levels in heart and almost is not expressed in lung tissue in mice (14). Due to Kv1.1 alpha subunit protein highly expressed in neural tissue, its presence and function are established in the vagal and phrenic nerves. The vagus nerve, the primary source of parasympathetic input to the heart, may contribute to Kv1.1-associated cardiac bradyarrhythmias in Kcna1^−/−^ mice (12). In addition, blood gas instability triggers a compensatory effects that may increase respiratory drive (26). So, *Kcna1 gene* may be a powerful candidate gene for Human SUDEP.

### KD Treatment Extends Longevity and Decreases Seizure Progression in Kcna1^−/−^ Mouse

Previous studies have shown that KD treatment exhibits a higher seizure threshold and a lower mortality rate in multiple models of inducible seizures as compared to mice fed a standard diet (SD) (6, 29). KD treatment also reduces seizure numbers and hippocampal hyper-excitability in Kcna1^−/−^ mice at ~P35–45 (20, 24, 30).

Recent studies have also shown that compared to SD-treated control mice, KD treatment reliably extends longevity of Kcna1^−/−^ mice. Kcna1^−/−^ mice experience sudden unexpected death at P47 while KD treated Kcna1^−/−^ mice prolong to P70 (13). This could be explained by the facts as following: (1) Kcna1^−/−^ mice exhibit a daily seizure frequency of 12 while KD treated mice experienced daily seizure frequency of 4 at P40–45 (13). KD treatment profoundly reduces seizure numbers when compared to control Kcna1^−/−^ mice at P30–39 and P40–49, respectively. (2) KD-treated mice display fewer severe seizure phenotypes when compared to control Kcna1^−/−^ mice age-matched at P25–29 and P30–39, respectively. Nonetheless, the seizure characterization of KD treated mice feature a similarity to control Kcna1^−/−^ mice at P50–59 (17) and KD treatment fails to block a terminal GTC-driven sudden death.

### KD Treatment Improves Rest Deficiency Accumulation in Kcna1^−/−^ Mouse

Studies have shown that KD treatment significantly enhances rest time in Kcna1^−/−^ mice. However, during the first 2 weeks, there is no difference in rest time in Kcna1^−/−^ mice between treatment with KD and SD. Compared to WT mice, there is a significantly reduction in rest time with age in Kcna1^−/−^ mice after P38. But there is a protection in rest time which became apparent in KD treated mice from P38. Interestingly, once KD treated mice reach P50, there is no difference in rest time between P50 and older age groups. In addition, when rest time declines ~30–40% less than WT mice, Kcna1^−/−^mice will die within 24 days and KD treated Kcna1^−/−^mice will die within 12 days. Taken together, these data suggest that the rest time in Kcna1^−/−^ mice decrease with age (18). In contrast, KD treatment offers protective effects on age-related rest reduction in Kcna1^−/−^ mice. However, rest time does not play a role in either Kcna1^−/−^ or KD treated mice when it approaches death.

The rest deficiencies are involved in sudden death in Kcna1^−/−^ and KD treated Kcna1^−/−^ mice (18). KD treated Kcna1^−/−^ mice experience more rest deficiency as compared to Kcna1^−/−^ mice. However, the ratio of rest deficiency before death is similar between Kcna1^−/−^ and KD treated Kcna1^−/−^ mice when normalized to their lifespan (18). The results suggest that both groups experience similar overall amounts of rest deficiency when normalized to their longevity.

The mortality in both Kcna1^−/−^ and KD treated Kcna1^−/−^ mice occurs during a predictable window. Acute levels of rest time before death are not sensitive to the timing of death, but the chronic accumulation of rest deficiency before death are sensitive to the timing of death. The chronic accumulation of rest deficiency over the final 10 and 15 days contributes to 58–75% of deaths in both Kcna1^−/−^ and KD treated Kcna1^−/−^ mice, respectively (18). These data indicate that a predictable time window in animal model might be converted into clinical data for prevention of SUDEP in individuals.

### KD Treatment in Gender and Age of Kcna1^−/−^ Mouse

Studies have shown that the gender can affect lifespans of Kcna1^−/−^mice at KD treatment initiation (13). Male and female mice with deletion of *Kcna1* gene display similar longevity in their life. However, KD-fed male mice have shorter lifespans than their female counterparts, and there is a trend toward a higher seizure frequency in KD-fed male mice than their female counterparts. Furthermore, the age of KD initiation also can affect longevity of Kcna1^−/−^ mice. Kcna1^−/−^ mice start on KD treatment at P35 have shorter lifespans than those start at PD25 (13). Taken together, the findings indicate that the KD treatment can hinder disease progression and sudden, unexpected death in Kcna1^−/−^ mice, and the protective effects by KD treatment are associated with gender and age at KD initiation.

### KD Treatment Increases β-hydroxybutyrate Levels in Kcna1^−/−^ Mouse

β-hydroxybutyrate (BHB) has been considered as the main indicator of the therapeutic benefits of KD treatment (31). BHB levels in plasma are generally taken as indicator for ketosis, but it interacts with classic antiepileptic drugs (AEDs) (32). Compared to both Kcna1^−/−^ mice and WT controls, KD treatment has higher blood BHB levels throughout life. In addition, KD treatment has lower glucose levels in WT control than Kcna1^−/−^ mice (17). Of interest, while all KD treatment groups show elevations in blood BHB levels, male Kcna1^−/−^ mice reveal significantly lower BHB concentrations as compared to female counterparts (13). It suggests that the differences in longevity of KD treated mice might be associated with gender correlated with BHB levels. Based upon these research results, it suggests that higher levels of BHB may control seizures more effectively in female than male mice (4). Further studies are needed to explore why BHB levels affect longevity in KD treated mice (33).

### Mechanisms of KD Treatment in Control Seizure and SUDEP

There are few studies about the mechanisms of the KD action in Kcna1^−/−^ mice for improving the seizures in SUDEP. Recently, researchers examined the relationship between gut microbiota and anti-seizure effects of the KD in two mice models (one of them was Kcna1^−/−^ mice) (34). They showed that KD-mediated seizure protection can alter the composition of gut microbiota, and they concluded that the protection of the KD on epileptic seizures is regulated by the gut microbiome through modulation of hippocampal GABA/glutamate ratios (34). Another important findings suggested that brain *PPAR*γ*2*, one isoform of peroxisome proliferator activated receptors (PPAR), had beneficial neuroprotection and anti-seizure effects in response to epilepsy. These findings indicated that *PPAR*γ*2* play an important role for KD therapy in Kcna1^−/−^ mice (35). Besides, Ketone bodies, the products of fatty acid oxidation by the high-fat KD in liver, may be as a fundamental mediator, exert anti-seizure effects in Kcna1^−/−^ mice through activation of mitochondrial permeability transition (30).

The mechanisms of the KD action in Kcna1^−/−^ mice, have also been performed in other animal models or Human epilepsy. The metabolic changes in the blood and cerebrospinal fluid were often considered to be the main factors of reducing seizures for KD therapy. The decrease in glucose levels and increase in KB were involved in KD mechanisms. In addition, improvement of mitochondria function leads to energy reserve, then activates ATP-sensitive potassium channels, which would stabilize synapse and attenuate neuronal excitability (36). Moreover, adenosine may be play a crucial role associated with this procession via an adenosine-dependent DNA methylation modulation (37). Furthermore, other researchers have showed that the KD has a protection against oxidative stress and mitochondrial dysfunction derived from epilepsy. These effects might be caused by diminishing reactive oxygen species and raise the mitochondrial uncoupling protein activity and the biosynthesis of glutathione (14, 38, 39).

The hippocampus might be one of the critical region of KD in Kcna1^−/−^ mice. The study suggested that KD treatment improves CA3-generated pathologic oscillations by decreasing mossy fiber synapses excitability (24).

### Perspective of KD Treatment Associated With SUDEP

Although studies have demonstrated that KD treatment improves the quality of life in Kcna1^−/−^ mice from different aspects, which might be associated with SUDEP, there are still some questions required to discuss.

The advantage of KD treatment in therapy of epilepsy has been recognized in pharmacological research and clinical application. However, KD treatment also reveals negative effects during treatment periods (40). Common negative effects include dehydration, hypoglycemia, growth alterations, gastrointestinal upset, hyperlipidemia, nephrolithiasis, and deficiency in vitamins, minerals, and electrolytes (41, 42). Therefore, it is not recommended for KD treatment more than 2 or 3 years. Interestingly, 60% of the patients with KD treatment display hyperlipidemia (43). These adverse effects can be controlled with help by nutritionist or medication.

Kcna1^−/−^ mouse is a clinically relevant animal model of SUDEP among the few models available. It manifests severe SRS, which relates to SUDEP (13). However, we should keep in mind that there is no one animal model mimic SUDEP in Human. Several cellular mechanisms have been suggested for KD treatment, including activation of ATP-sensitive potassium channels, inhibition of glycolysis, and disturbance of glutamatergic synaptic transmission (44). The molecular mechanisms of underlying the KD treatment have been remained unclear. In future, researchers are required to focus particular on the underlying mechanisms of genetic basis of SUDEP as well as KD treatment for SUDEP.

Although emerging data acquired from animal experiments, preclinical and clinic studies regarding the relationship between KD treatment and SUDEP are less developed. Future studies are needed to integrate preclinical and clinical studies to explore the risk factors of SUDEP, and thus hopefully open a new window for proactive and preventative treatment strategies of SUDEP in high-risk individuals (18).

Previous studies focused on the association between SUDEP and Kcna1^−/−^ mice in different aspects. There is still lack of systematic studies on the mechanisms of SUDEP in Kcna1^−/−^ mice in detail, and basically few research on Human SUDEP and KD. Therefore, further studies on the association among Human SUDEP, Kcna1^−/−^ mice and KD should be strengthened, so as to serve the clinical Human SUDEP more efficiently.

## Author Contributions

The overall review design was conceived and supervised by X-PC. YR, JC, CL, CJ, PL, YW, and X-PC wrote different parts of the review. All authors read and approved the final manuscript.

### Conflict of Interest Statement

The authors declare that the research was conducted in the absence of any commercial or financial relationships that could be construed as a potential conflict of interest.
